# “I noticed that when I have a good supervisor, it can make a Lot of difference.” A Qualitative Study on Guidance of Employees with a Work Disability to Improve Sustainable Employability

**DOI:** 10.1007/s10926-022-10063-6

**Published:** 2022-09-06

**Authors:** R. Schaap, V. A. Stevels, M. S. de Wolff, A. Hazelzet, J. R. Anema, P. Coenen

**Affiliations:** 1 Department of Public and Occupational Health, Amsterdam UMC, Vrije Universiteit Amsterdam, Amsterdam Public Health research institute, Amsterdam, The Netherlands; 2 Sustainable Productivity and Employability, Netherlands Organization for Applied Scientific Research TNO, Leiden, The Netherlands

**Keywords:** Employees, Work disability, Supervisors, Sustainable employability, Qualitative study

## Abstract

**Purpose:**

For employees with a work disability adequate daily guidance from supervisors is key for sustainable employability. Supervisors often lack expertise to guide this group of employees. Mentorwijs (literal translation: Mentorwise) is a training for supervisors to improve the guidance of employees with a work disability. The aim of this study was to investigate the experiences of employees with a work disability regarding: (1) the guidance from their supervisors (who followed the Mentorwijs training), (2) which differences they notice in the guidance due to the Mentorwijs training, and (3) what kind of aspects they consider important in their guidance to achieve sustainable employability.

**Methods:**

A qualitative study was performed with semi-structured (group) interviews among twenty-one employees with a work disability. Thematic analysis was performed to analyze the data.

**Results:**

Themes that followed from the interviews were: (1) work tasks and conditions can facilitate or hinder sustainable employability: (2) relationships among employees and with supervisors can affect sustainable employability; (3) a desire for new opportunities and challenges; and (4) a need for supervisor skills to facilitate sustainable employability, i.e. appreciation, availability of help, dealing with problems, listening, attitude and communication. According to employees, changes were mainly noticed in supervisor skills.

**Conclusions:**

Employees with a work disability were very satisfied with the guidance of supervisors who followed the Mentorwijs training. To improve sustainable employability, training of supervisors should focus more on adequate work conditions, providing employees opportunities to learn new work tasks and improving supervisors’ skills regarding appreciation, attitude and communication.

**Supplementary Information:**

The online version contains supplementary material available at 10.1007/s10926-022-10063-6.

## Background

Work is generally considered good for one’s health, because it can offer financial independence, which in turn reduces psychological distress, and improves physical and psychosocial well-being [1, 2]. In contrast, those unemployed and with insecure work have higher mortality rates and poorer physical and mental health than people with a job [1, 3]. In certain groups, unemployment and job insecurity are more prevalent than in others. One of those groups are employees with a work disability that are employed in supported workplaces and/or in the regular labor market. This can include people with a (mild) intellectual disability, psychological disability, physical disability, (very) low level of education and/or learning delay [4]. In the Netherlands, there were in 2019 more than 800 thousand persons between 15 and 65 years old who were prevented from obtaining or maintaining sustainable work due to a long-term illness, a disorder, or disability [5]. About 45–50% of these people had a paid job, while the remainder received social insurance benefits [5]. Social insurance benefits place a significant financial burden on society and being unemployed has, as mentioned earlier, negative health consequences. Therefore, it is important that employees with a work disability find work and maintain employed.

For employees with a disability, it is hard to find a job [6, 7]. Moreover, when they have a job, employees with a work disability less frequently have a permanent contract than employees without a work disability [8]. Studies on the reasons why companies do not hire employees with a work disability showed that supervisors believe that this group of employees is less productive and more absent, and therefore supervisors prefer someone without work disabilities with equal suitability [4, 9, 10]. Improving sustainable employability is a way to ensure that employees with a work disability will find work and maintain employed [11]. Sustainable employability is defined as employee’s ability to contribute through their work, while learning skills, maintaining good health and well-being throughout their working life [12, 13]. Sustainable employability consists of four core components: health, productivity, valuable work, and long-term perspective [12]. For employees with a work disability, optimal guidance from their supervisor by focusing on these components is key for sustainable employability [4]. Research shows that training supervisors in providing the right type of guidance can reduce absenteeism and promote reintegration of employees with a work disability, and improve sustainable employability [14]. When supervising employees with a work disability, a supervisor must for instance, set clear expectations and motivate the employee by providing good examples [4]. However, unfortunately supervisors often lack the expertise to adequately guide employees with a work disability [15]. They may have negative perceptions and attitudes and little knowledge about employees with a work disability and the guidance they need [16–18]. Therefore, guidance of supervisors needs to be improved to increase sustainable employability of employees with a work disability [4].

Mentorwijs (literal translation in English: Mentorwise, which refers to the supervisor who takes the role of mentor) is a training that has been developed for supervisors to better guide employees with a work disability. The aim of the training is to develop and strengthen the knowledge, attitudes, and skills for adequate guidance of employees with a work disability. Supervisors, who have completed the Mentorwijs training are generally positive about the training [4]. However, it is unknown what the experiences of employees are regarding the guidance of supervisors who have followed the Mentorwijs training and what kind of aspects they find important for their sustainable employability. Such information could provide relevant insights for those supervising employees with a work disability, in the context of Mentorwijs and beyond. Therefore, a qualitative study was conducted to answer the following research question: What are the experiences of employees with a work disability regarding (1) the guidance of supervisors (who followed the Mentorwijs training), (2) which differences they notice in the guidance due to the Mentorwijs training, and (3) what kind of aspects they consider important in their guidance to achieve sustainable employability?

## Methods

### Study Design

In this qualitative study, semi-structured (group) interviews were held with employees with a work disability to obtain insight into their experiences about the guidance of supervisors at the workplace. The Medical Ethics Committee of the VU University Medical Center approved the study protocol and decided that the Medical Research Involving Human Subjects Act does not apply to this study (reference no. 2019.239). This study, which is part of a larger study on the effectiveness of the Mentorwijs training, is also registered in the Dutch Trial Register [19]. The COREQ (Consolidated criteria for reporting qualitative research) checklist was used to conduct and report this study [20]. All participants provided written informed consent before participating in the study.

### Mentorwijs Training

The Mentorwijs training focuses on supervisors in regular labor organizations and consists of five meetings of 2.5 hours, each with specific learning objectives. The training is face-to-face with a combination of theory and practice, with ample opportunity for supervisors to interact and share experiences from their daily practice. The training focuses on 1) developing knowledge on type of work disabilities and possibilities for support or adjustments at the workplace for employees with a work disability, 2) building an open and involved attitude of supervisors to enhance the autonomy of employees, 3) strengthening specific skills, such as applying different leadership styles and skills for communication, and 4) developing and strengthening knowledge, attitudes and skills to increase the self-efficacy of supervisors regarding the guidance of employees with a work disability.

### Recruitment

Supervisors who had followed the Mentorwijs training were approached to help recruit employees with a work disability who were direct reports of these supervisors, using a convenience sampling approach. After signing informed consent, employees completed a short questionnaire wherein they answered questions regarding their 1) age, gender and education, 2) type of work and organization, and 3) type of disability. Employees could also indicate if they agreed to be approached for an interview. Supervisors of employees that agreed to be approached for an interview were asked by the researchers to schedule an interview. The interviews took place at the workplace, as this was a familiar environment for the employees, making it easier to reach this target group. For each interview we aimed to recruit several employees, because this could stimulate discussion and portray multiple perspectives. Employees could also feel more comfortable in the presence of their colleagues, which could make them more inclined to participate. As a single supervisor typically supervised multiple employees, most employees could be interviewed as a group at the workplace. Due to our sampling strategy information of supervisors on how many employees refused to participate in an interview was difficult to determine.

### Data-collection

An interview guide was used to conduct semi-structured interviews. This interview guide consisted of topics with (sub) questions regarding: [1] job satisfaction, [2] guidance satisfaction, [3] change in guidance after the Mentorwijs training [4] employee’s satisfaction of the fit between knowledge and skills and the demands of the job, [5] confidence regarding performance of the job (self-efficacy), and [6] position in the company (Appendix 1). The interview guide was used to ensure that the same topics were discussed in every interview. The topics were based on important aspects for sustainable employability of this target group [4, 21]. The interviewers primarily asked about valuable work components and components for long-term sustainable employability [12]. This was done by asking employees for opinions about their work and work tasks and whether they see themselves working for a long time at the current company. Less emphasis was placed on the other components of sustainable employability (i.e. health and productivity), because the Mentorwijs training did not aim to improve the health and/or productivity of workers with a work disability. The training focused merely on the valuable work component and long-term perspective, such as job motivation and the fit between the job and the employee to increase the chance that employees with a work disability remain employed over a longer period of time.

Interviews were audio-recorded and conducted at the workplace between October 2019 and April 2021, at least 3 months after their supervisor completed the training. Interviews were only conducted with employees - the supervisor was not present, and employees were ensured that audio-recordings and transcripts were not shared with their supervisors. Prior to the interview, employees were informed about the aim of this research, but not about personal aims of the researchers. No relationship was established between the employees and the researchers prior to the study, and no repeat interviews were conducted. The interviews started by getting to know each other and asking the employees what kind of work they do. Interviews were conducted until data saturation occurred and lasted 20–40 min. Two female researchers were present at each interview. One researcher, who was experienced in conducting interviews, led the interview (RS), while the other researcher, who was less experienced, observed and asked additional questions when necessary (VS). RS is an occupational health researcher with previous experience in conducting interviews and qualitative research. VS is a Health and Life Sciences Bachelor student, who was trained in qualitative research and interviewing skills. There were differences in the social status and educational level between the researchers and employees. However, researchers aimed to create a safe environment, to ensure that employees felt comfortable. Using their training and experience in qualitative research with vulnerable populations they aimed to remain objective as possible and used clarifying questions to fully understand the answers of employees. No field notes were made during interviews, but every interview was evaluated, and results were considered in future interviews or in data-analysis.

### Data-analysis

To analyze the data, interviews were transcribed verbatim and transcripts were pseudomized by removing all identifiable information. The transcripts were coded inductive and iterative using ATLAS.ti 8., using an interpretative constructivist approach (i.e. focused on how people interpret reality and to understand how people see or experience the world) [22] to explore and understand the experiences of employees with a work disability. Thematic (content) analysis was used to analyze the data and identify themes using open coding, axial coding, and selective coding [22, 23]. First, one interview was independently open coded by two researchers (VS, RS), and the codes were compared for consistency. Conflicts were resolved and a common coding method was determined. Second, one researcher (VS) coded four interviews. Third, a consultation about the coding method between two researchers (VS, RS) took place, after which the remaining interviews were open coded by one researcher (VS). Forth, during axial coding all codes were discussed, and categories of codes were formed (VS, RS). Fifth, continuous consultation between the researchers (VS, RS, PC) and constant comparison took place to increase the reliability of the codes. New categories were created, renamed, merged and eventually visualized to obtain a clear overview of how the codes related to each other. Sixth, selective coding led to the formation of themes during a consensus meeting (VS, RS, PC). See Appendix 2 for the codebook. These themes were narratively described, to describe the experiences of employees with a work disability. Transcripts were not returned to the employees for comments and/or corrections, and no member check took place. Though, in all stages of the data-analyses the researchers critically reflected on the codes, categories and themes that emerged from the data, by checking the interpretations obtained in each phase and by going continuously back to the data. Themes were substantiated with relevant citations from the interviews (that were translated from Dutch into English). Data-analysis was performed in parallel with data-collection, hence researchers could decide whether data-saturation was reached based on the content of the interviews.

## Results

### Study Population

Interviews were held with twenty-one employees with a work disability whose supervisors followed the Mentorwijs training. It concerned ten interviews, of which seven were group interviews with two or more employees (up to four per interview) and three interviews with one employee. The interviewees consisted of seventeen men (81%) and four women, ranging between 20 to 61 years of age and with a lower (71%), middle (24%) or higher educational level (5%) (Table 1). Employees had a mild intellectual disability (23%), lower education level and/or learning delay (27%), psychological disability (23%), and/or a physical disability (14%). For some employees the disability was unknown (14%), as they were not aware of their disability or were not willing to answer this question. Employees with a work disability had various occupations within various companies, such as gardener (57%), production employee (19%), administrative employee (10%), kitchen worker (10%) or cleaner (5%).

**Table 1:** Characteristics of the study sample

	n = 21
**Age**	
Mean (SD)	41.5 (13.1)
Range	20–61
**Gender**	
Men	17 (81%)
Women	4 (19%)
**Educational level**	
Low	15 (71%)
Middle	5 (24%)
High	1 (5%)
**Disability**	
Low level of education and/or learning delay	6 (29%)
Mild intellectual disability	5 (24%)
Psychological disability	5 (24%)
Physical disability	3 (14%)
Unknown	3 (14%)
**Occupation**	
Gardener	12 (57%)
Production employee	4 (19%)
Administrative employee	2 (10%)
Kitchen employee	2 (10%)
Cleaner	1 (5%)

Various themes emerged from the interviews: work tasks and conditions can facilitate or hinder sustainable employability, relationships among employees and with supervisors can affect sustainable employability, a desire for new opportunities and challenges, and need for supervisor skills to facilitate sustainable employability. Results associated with these themes are described below.

### Work Tasks and Conditions can Facilitate or Hinder Sustainable Employability

Employees indicated many facilitators and barriers within their work and work tasks for sustainable employability. The most prominently facilitators mentioned were that work was considered fun, easy, and there was an enjoyable atmosphere. Employees also mentioned that there was no large workload, they had a lot of freedom in performing their work tasks (independently), and they wanted to do this work for a long time. In addition, they stated that their work tasks were diverse, not difficult, structured and often carried out independently:

*E14: “I am more drawn into my own, so when I know what to do, I go my own way. For some work tasks it is nice that they help me, but most tasks I can do myself”* (Man, 23 years).

Some employees also stated that adjustments were made at the workplace to facilitate performing their work tasks. For example, one worker mentioned that he could perform his work tasks step-by-step at its own pace. On the other hand, barriers for sustainable employability within work tasks were also mentioned. In contrast to employees that were positive about their work, others described that the work was often monotonous, boring and energy consuming. Some employees stated that work that required a lot of concentration was hard. They also mentioned they had to continue working outside, despite the bad weather conditions, or sometimes had a lot of work hours or had to work hard:

*E4: “They always say that we have to work hard. That is ridiculous, because they say we have to work hard but they also say we are employees with a work disability”* (Man, 59 years).

In addition, employees mentioned that they were not always satisfied with their working conditions. Some employees indicated that they did not have proper work clothes and insufficient breaks. Barriers within work tasks and working conditions resulted in needs; for example, that employees wanted to feel useful at work, have more responsibility, more variation in work tasks, more structure in the workplace, and perform work with societal relevance. Needs related to workings conditions involved proper work clothes and more breaks.

### Relationships Among Employees and with Supervisors can Affect Sustainable Employability

Employees also discussed their relationships with other employees and with their supervisor. Both positive and negative elements from these relationships were mentioned that could impact sustainable employability. Employees mentioned they were generally positive about relationships with their colleagues and that collaboration between colleagues went well. For example, an employee indicated that he has colleagues with a lot of experience, who help him well with his work tasks if these are too difficult. In addition, many employees spoke about the importance of equality in the workplace. Employees indicated they were seen as equal by their colleagues, and they were also treated equally by their supervisor. Employees with and without work disabilities were treated equally, as was said by an employee:

*E8: “Everyone is equal. Nobody is more than the rest.”* (Man, 49 years).

Employees also reported that there was little hierarchy between colleagues with the same occupation. For several employees, conflicts between colleagues therefore hardly occurred. They said that they were pleased that they had not experienced any conflicts with other colleagues:

*E13: “No, I never have them [conflicts]. Yes, that’s great.”* (Woman, 44 years).

Although most employees indicated that there was indeed equality at the workplace, this was not the case for every employee. Some colleagues considered themselves more important than others:

*E14: “There is always a distinction between the employees from the office and employees from the production [….]. You have to do the work together, if we [employees from production] don’t do anything, then they [employees from the office] can do what they want, but then nothing happens”* (Man, 23 years).

Another employee indicated that conflicts with his supervisor sometimes occurred, with unpleasant working conditions being a reason for such conflicts. Other employees also mentioned negative elements of relationships at the workplace. For example, one employee indicated that there was a lot of gossip at work, which he did not like, and which resulted in a poor relationship with his colleagues. Other employees said that there were colleagues they did not like or irritations between employees occurred, which were then resolved by the supervisor.

Besides the relationships among employees, interviewees also talked about the relationship with their supervisor. Some employees mentioned that conflicts were relatively quickly resolved by talking about the matter. Such conversations were often initiated by the supervisor. It was also mentioned by one employee that there was a lot of understanding for his work disability from the supervisor. In contrast, another employee felt he was treated like a child and even hated his supervisor:

*E15: “He thinks he is powerful, that can simply be said. Just a cocky bastard. As soon as things go well it’s all good, but when things go wrong, he will yell at someone. But the mistake is never his fault.”* (Man, 20 years).

According to one employee, the relationship with their supervisor had positively changed because of the Mentorwijs training. As a result of the training they communicated more, considered each other in a better way and worked more together:

*E14: “First, everyone was on his own island and now it is more like he says: a little more communication and a little more cooperation and more consideration for others.”* (Man, 23 years).

### A Desire for New Opportunities and Challenges

Employees discussed the desire for challenges in their work tasks and new opportunities to learn new work tasks, to have variety in work tasks, and to get the opportunity to further develop themselves in performing their work tasks. These desires also prompted questions about the current possibilities and opportunities to learn new skills and tasks. Some employees mentioned that work was educational, challenging, there were opportunities to learn new work tasks, to make mistakes, and to get opportunities to grow:

*E20: “I have been working in the kitchen for a while, and now I received training from the organization, and over the years I have been given more responsibility.”* (Man, 33 years).

An employee also indicated that it is nice to learn new things step by step. However, several employees said that these learning moments were scarce and that they wanted them more often. This showed that the desire for new opportunities and challenges is greater than the current supervisors or employers could and/or wanted to provide:

*E19: “Yes, you can follow a training. I already asked my supervisor a few times, but I still haven’t heard from that. I still don’t know if anything will ever go through, I just want to be able to work my way up.”* (Man, 31 years).

### Need for Supervisor Skills to Facilitate Sustainable Employability

During the interviews, various skills (both positive and negative) of a supervisor were discussed, what employees would like to see in the skills of their supervisor and what role they felt the Mentorwijs training had played in this. Most employees were satisfied with the guidance they received at the workplace, felt that no changes were necessary, and did not criticize their supervisor. However, not all employees were positive about the guidance and indicated that there was room for improvement.

#### Communication

One skill of a supervisor that was mentioned by each employee was communication. Many employees indicated that their supervisor had a clear and pleasant way of speaking. In addition, several employees indicated that they received clear explanations regarding work tasks. Clear communication was one point that made employees satisfied with the guidance they received at work. On the other hand, communication from the supervisor did not always go well according to some employees, as there was occasional contradictory or unpleasant communication. Some employees also indicated that a supervisor did not or not properly fulfil his promises to provide new work tasks or new work clothes:

*E5: “We often said: ‘when do we get other clothes?’ And then it was: ‘yes it comes, it comes.’ We are now two years later and we still have the same clothes.”* (Woman, 21 years).

One employee also stated that he did not like it when the supervisor not directly communicates with him, but communicated with others about his work functioning. Several employees also stated that they had little contact with their supervisor:

*E2: “I only see him [supervisor] in the morning at the workplace and I don’t see him any further.“* (Man, 54 years).

Employees expressed different desires about the communication with their supervisor. For example, employees would like to talk with their supervisor now and then. Other employees desired a clearer explanation of their work tasks, because sometimes it was unclear how to perform their work. According to some employees, the Mentorwijs training had changed the communication of their supervisors. An employee mentioned that his supervisor communicated better.

#### Attitude

Employees were, in general, satisfied with the attitude of their supervisors towards them. What was mentioned most regarding this skill and what employees were very satisfied with when it comes to their guidance, was that employees’ opinions were taken seriously:

*E9: “You wouldn’t say it because we all have a disability, but we are simply taken seriously.”* (Man, 53 years).

In addition, several employees indicated that their supervisors were friendly, reliable and considerate to employees, and that they trusted the employee in that they performed their work tasks well. Negative experiences of employees were that some indicated that their supervisor had a negative attitude. Even though employees were generally satisfied with the attitude of supervisors, some employees with the same supervisor indicated the following areas for improvement for their supervisor: they would want their supervisor to give them more autonomy, be more considerate and more patient, not treat them as children, trust them more, and take them more seriously. These employees were, in contrast to most of the other employees, not satisfied with their supervisor and many aspects of the guidance.

According to some employees, attitudes of supervisors had changed positively due to the Mentorwijs training. These employees were therefore very pleased that their supervisors followed the training. For example, an employee mentioned that his supervisor had become more relaxed, and another employee stated that supervisors who followed the training were very serious about the supervision. A change that was also noticed by some employees was that the supervisor kept a closer eye on the employee, and they talked and collaborated more with their supervisor when something was unclear.

#### Listening

Many employees stated that their supervisor listened carefully:

*E1: “She [supervisor] also listens well. So the moment I say that it doesn’t work well, she can also take that into account”* (Man, 30 years).

In contrast, some other employees mentioned that their supervisor was not listening well to their opinions or stated that a supervisor cut off criticism and that employees had little to say. They would like their supervisor to listen more:

*E5: “I mean I am not a 12-year-old child. It would be nice if they listen more to us”* (Woman, 21 years).

#### Dealing with Problems

Employees also described how they, as employees, deal with problems at the workplace. It became clear that when employees had a problem, they almost always went to their supervisor to discuss these problems. A problem was often picked up by the supervisor. For example, an employee described that he failed to complete his work tasks and was frustrated about this, but that his supervisor helped him to calm down:

*E16: “Then they just try to say ‘yes there’s no point in getting mad’. They say ‘just stay calm and then it will automatically be alright’.”* (Man, 36 years).

Most employees stated that supervisors were available to talk about problems. However, some employees were not satisfied, as their problems were not always addressed in a timely matter. Some employees stated that they wanted a supervisor that is willing to help employees with their problems.

#### Availability of Help

Employees also talked about the availability of help from supervisors. The majority was satisfied with the available help, as in almost every interview it was indicated that asking questions was always possible:

*E16: “I always notice that if I have a question and they [supervisor] are in the office, I walk to the office and then I ask: ‘would you like to help?’”* (Man, 36 years).

This was an important reason for employees being satisfied with the guidance that they receive, because employees were happy to have the opportunity to receive help and that supervisors notice when employees need help. However, some employees stated that they needed to initiate asking for help. Moreover, an employee described that, despite the possibility to always ask questions, the supervisor had little time for the employee. Another employee said that due to pressure at work the supervisor was sometimes unable to ask questions when he did not understand his work tasks:

*E7: “Sometimes he says: ’not now, can you come back later? I’m busy or I have to go to a meeting ‘. Then I have to wait.”* (Woman, 61 years).

Planned meetings between the supervisor and employee sometimes had been rescheduled due to a lack of time from the supervisor. Employees indicated that they would like their supervisor to always be available for questions and that they would like their supervisor to be more present in the workplace.

#### Appreciation

Appreciation was another skill that was regularly mentioned during the interviews. Employees indicated that they received appreciation and compliments for their work, and that compliments from supervisors gave them more motivation to work. One employee indicated that they received more compliments after the training. On the other hand, some employees mentioned that their supervisor showed little appreciation for their work because they received almost no compliment.

*E5: “We walk like 36 or 40 hours per week, only by foot, walking, walking, walking, and then it’s not even: ‘guys you are doing a good job’.”* (Woman, 21 years).

#### “Mentorwijs”

A number of abovementioned skills have changed and improved among supervisors by participating in the Mentorwijs training. Employees mentioned in the interviews that there was a difference in skills after supervisors followed the training, but it was difficult for employees to identify what this difference was. Employees also remained satisfied with the guidance of their supervisors after the training and, according to one employee, the supervisor said that he had learned which points he can improve on himself. However, most employees did not notice any difference in the guidance of supervisors after the training.

## Discussion

We investigated the experiences of employees with a work disability about the guidance they receive from supervisors (who followed the Mentorwijs training), whether they notice differences in the guidance due to the Mentorwijs training, and what kind of aspects were important in the guidance for their sustainable employability. In general, employees enjoyed their work, but work tasks were sometimes not challenging enough, and they wanted more appreciation and compliments from their supervisor. Main reasons for satisfaction about the guidance were that help was often available, their opinions were taken seriously, and equality in the workplace. Other employees were dissatisfied, mainly because they wanted their supervisor to give them more autonomy, to be more considerate, and trust them more. In several areas, the satisfaction of work and guidance of supervisors can be further increased, which may also increase sustainable employability of employees with a work disability. These areas will be discussed below, as education for supervisors, such as the Mentorwijs training, could help supervisors to learn about and implement these elements in their daily practice.

### Interpretation of the Findings

#### Working Conditions and Working Relations

Working conditions were not always pleasant according to employees with a work disability in this study. As mentioned earlier, employees with a work disability more often have a job insecurity (e.g. a flexible contract) than people without work disabilities [8]. From literature, it is known that a supervisor is more inclined to invest in an employee with a permanent contract [24]. This could be the reason why things like the right work clothing, but also training opportunities, were not always available for some employees with a work disability in this study. The difference between permanent and flexible contracts will therefore only widen the gap between employees with a work disability and employees without work disabilities [24], which can ultimately lead to reduced job satisfaction and sustainable employability. Another issue is that, although employees enjoyed their work, they also indicated it was sometimes not challenging enough. A key element of the Mentorwijs training is to ensure that employees enjoy going to work by strengthening their autonomy and not be too protective with them. Supervisors of employees with a work disability are therefore urged to provide good working conditions, including varying tasks and opportunities for growth, as will be discussed in the paragraph about opportunities and challenges.

Most employees were positive about relationships at the workplace, as they were treated equally and there was little hierarchy. This is also an important aspect in the Mentorwijs training, as supervisors learned to ensure equality at the workplace and to pay attention to possible frictions among employees. Social relationships at the workplace are known to increase job satisfaction [17], as being recognized and accepted contributes to the feeling of social inclusion [25]. However, not all employees experienced that their relationships were positive, as some felt being treated unequal or due to unpleasant communication or conflicts about work tasks or conditions with supervisors and/or colleagues. The latter was also found in another study, where employees who perceived their working conditions unpleasant, believed they were treated differently compared to their colleagues [25]. Therefore, open and equal communication between employees and supervisors about problems or possible adjustments to work tasks and conditions appears important [21, 25]. This may lead to a better work climate and more positive relationships [21, 25], which was also experienced by employees in this study.

#### Opportunities and Challenges

To create opportunities for development and to find challenges for the employees is part of the Mentorwijs training. However, one of the desires employees with a work disability had in this study was to learn new work tasks and to get the opportunity to develop themselves. This finding is supported by existing literature; for example a review showing consistent evidence that the opportunity for personal growth and development increases job satisfaction [17]. Another study on the experiences of employees with a work disability concluded that the feeling of being valued depends on the extent to which employees are provided with opportunities that enable personal development [25]. This increases the valuable work component of sustainable employability, and may therefore also improve sustainable employability of employees with a work disability. This is in line with studies that showed that having the possibility to and learn new skills and work tasks may increase sustainable employability among employees with a work disability [21, 26].

#### Skills of the Supervisor

Important skills of the supervisor that, according to the interviewees, could improve the guidance were communication, attitude, listening, dealing with problems, availability of help and appreciation. During the interviews, it became clear that some employees noticed positive changes in the skills among supervisors who followed the Mentorwijs training, which aimed to improve supervisors’ knowledge, attitude, and skills [4]. Positive changes were, among other things, improved communication between the supervisor and employee, receiving compliments, and that the supervisor and employee were more considerate to each other. These skills are part of the Mentorwijs training, as supervisors learn about different leadership styles, communication techniques, how to give feedback and how their own attitude may affect the employability of employees. However, most employees did not have a strong opinion about the effect of the Mentorwijs training for their supervisor, as they did not notice any (negative or positive) difference in the guidance after the training. Our findings therefore do not provide strong evidence that the Mentorwijs training did change the guidance of employees with a work disability. Further research must provide more insight into the extent to which the Mentorwijs training improves the guidance of employees with a work disability.

In general, employees felt that their supervisors communicated clearly, but sometimes there was contradictory or unpleasant communication. Communication from the supervisor to employees with a work disability must be clear and understandable, as unclear communication could lead to conflicts between supervisors and employees in case employees cannot meet the supervisors’ expectations [21]. The challenge for supervisors is to set clear expectations and give concrete instructions about work tasks. This is in line with previous research showing that good and open communication between the supervisor and the employee is important to discuss adjustments of work tasks or in the work environment [21, 25], as this may increase job satisfaction and thereby sustainable employability of employees with a work disability [27].

Employees in our study generally spoke positively about the attitude of supervisors. It is important that supervisors maintain this attitude because research showed that negative attitudes from the supervisor to employees has a negative influence on sustainable employability [28]. However, employees that we interviewed indicated that the attitude of their supervisor was not always good. An earlier study showed that supervisors tend to have negative attitudes about employees with a work disability, which is mostly caused by the concern that employees would be less productive [9, 10, 29]. This could lead to supervisors closely observing employees on their work performance. As was described by employees in our study, this can negatively impact employee’s satisfaction as employees described that they wanted their supervisors to give them more autonomy, trust them more and take them more seriously. Moreover, research on U.S. veterans and their supervisors showed that when supervisors’ attitudes toward veterans improve, the veterans’ sleep and health outcomes also improve [30]. Although this concerns a different target group, it does show how much effect a supervisor’s attitude can have on employees.

Employees in our study also found compliments and appreciation important, and they wanted more appreciation for the work they were doing. Earlier research found that employees needed compliments and appreciation from their supervisors for the work tasks they performed, while they also liked to receive compliments and appreciation from their colleagues in similar occupations [31]. This increases the feeling of being valued, which can lead to higher job satisfaction [31, 32]. Receiving more compliments and appreciation from supervisors, but also from colleagues, could therefore increase sustainable employability of employees with a work disability.

### Strengths and Limitations

Several strengths and limitations were identified in this study. A strength of this study was that the interviews took place in different types of industries, resulting in a sample of employees from different occupations. In addition, employees had various work disabilities and there was a wide age range. The variation in industries, occupations, work disabilities, and the broad age range increased the generalizability of the results. However, due to using convenience sampling, our sample is not necessarily representative for the group of employees with work disability. Moreover, employees were recruited by their supervisors and interviews were conducted at the workplace. Despite the actions we have taken to ensure that employees felt comfortable to be fully transparent about their thoughts and feelings, there is a possibility they may not have felt comfortable to talk openly about the guidance. Another limitation is that it was difficult to interview employees about their work and guidance, because sometimes the questions were not understood by employees, the answers were short or unclear and the question for clarification or underlying reasons of an answer could not always be answered. Moreover, transcripts were not returned to the employees and no member check too place. To increase the credibility of the results we conducted the data-collection and data-analysis with multiple researchers. Another strength of this study is that the interviews were conducted at least 3 months after supervisors completed the Mentorwijs training. We used this time frame to be more assured that changes have taken place in the guidance of employees due to the Mentorwijs training. However, supervisors may need more time to change the guidance of employees with a work disability. Also, due to the qualitative study design, changes are not necessarily causally linked with the Mentorwijs training. For example, behavioural changes may also be caused by changes in the organization’s broader climate and culture. To determine a causal relationship between the training and changes in the guidance other, more quantitative controlled, study designs are needed in future research. Employees also found it difficult to notice changes due to the Mentorwijs training. During the interviews it became clear that some employees were not even aware that their supervisor had completed the Mentorwijs training and others had not been employed long enough to notice a clear difference between the guidance before and after the training. Besides, it remains the question whether employees with a work disability, for example a mild mental disability or learning delay, can sufficiently reflect on, notice and name possible changes. It is therefore possible that changes in guidance because of the Mentorwijs training have taken place, but not have been noticed by employees with a work disability. Despite these difficulties, attempts have been made to obtain information from employees with a work disability. For example, the questions were easily formulated, the interviews took place in a familiar environment, and in most of the interviews (7 out of 10) employees were together with at least one colleague.

### Implications for Research and Practice

Further research on employees with a work disability should focus on how the working environment can be improved, and how supervisors can be convinced of hiring and investing in employees with a work disability. Further research should also focus on how supervisors can recognize the desires of employees to learn new skills and/or work tasks, how to provide these opportunities, and how they can create a safe environment where there is room for employees to make mistakes. This could facilitate a work climate wherein employees can informally learn and develop themselves, which likely increases the sustainable employability [21]. However, for supervisors to create a learning work climate, it is important they receive support at organizational level – e.g. that organizations have policies on training and development, or supporting technologies to facilitate learning [33]. Moreover, from this study, it is not clear whether the size of the company or type of workplaces influences the guidance of supervisors, while research shows that this could have an effect on employment [34]. Studies that examined the differences between supported and sheltered workplaces showed that employees in supported workplaces are more satisfied with their job than employees in sheltered workplaces [17]. According to the Dutch system, sheltered workplaces create jobs for employees with a work disability that are not able to work in the regular labor market. Supported workplaces are jobs for employees with a work disability in the regular labor market, but wherein these employees receive support related to their disability (e.g. job coaching, training). Therefore, it is important to do more research on the size and type of workplaces of employees with a work disability, as this could also influence the guidance they receive from supervisors. At last, this research focuses on the guidance of employees with a work disability in relation to sustainable employability. However, the private situation of the employee also plays a major role in their employability [21], as problems (e.g. unhealthy living conditions or financial problems) in the private situation may have direct negative effects on the employability of workers. Therefore, to adequately improve sustainable employability, future research should also focus on how supervisors can deal with problems in the private situation that affect the employability of employees with a work disability.

This research also showed that there were some points for improvement for supervisors about the guidance of employees with a work disability, namely providing challenges in work tasks and opportunities for growth, appreciation and giving compliments to employees, investing in employees’ autonomy, that employees are taking seriously, and improve communication of supervisors. An addition to the training could, for example, be how supervisors should deal with employees who want more challenge in their work tasks and how supervisors can better distribute their attention and time so that employees can receive more personal attention. In addition, the training can emphasize that giving compliments and expressing appreciation is extremely important for employees and that it is important to have good and open communication with employees to facilitate adequate adjustments to work tasks and conditions. How to deal with these points of improvement can be applied in Mentorwijs or other related trainings for supervisors of employees with a work disability. Improving the training can increase employees’ satisfaction about their job and guidance, after their supervisors have completed this training, and thus improve sustainable employability.

## Conclusions

Our findings indicate that employees were, in general, very satisfied with the guidance of supervisors who followed the Mentorwijs training, and believed that not much needed to be changed in their guidance. Possibly because of this, changes in the guidance were hardly noticed by many employees. Also, because they may not be aware of the exact content of the Mentorwijs training. Despite this, several aspects in the guidance of supervisors were identified that affect the sustainable employability of employees with a work disability. To improve sustainable employability of employees with a work disability, training of supervisors in guidance of these employees should focus more on adequate work conditions, opportunities for development and improving supervisors’ skills regarding appreciation, attitude and communication.

## Electronic supplementary material

Below is the link to the electronic supplementary material.

Supplementary Material 1

Supplementary Material 2

## Data Availability

The data consist of transcripts of interviews (in Dutch) and are available from the corresponding author on reasonable request.
