# Supplementary material for: “I noticed that when I have a good supervisor, it can make a Lot of difference.” A Qualitative Study on Guidance of Employees with a Work Disability to Improve Sustainable Employability
Source: J Occup Rehabil. 2022 Sep 6;33(1):201–12. doi: 10.1007/s10926-022-10063-6 (PMC10025227; doi:10.1007/s10926-022-10063-6)
Supplement: Supplementary file 1 — Supplementary Material 1 [file 10926_2022_10063_MOESM1_ESM.docx]

**Appendix 1: Interview guide**

**Questions**

1. What kind of work do you do? Or: What kind of work tasks do you have?

*Topic 1: Job satisfaction*

1. What do you think of your work?
   1. Do you like this job?
   2. What do you like or do not like about this job?
   3. Do you enjoy going to work?
   4. Would you like to do this work for a longer period of time?

*Topic 2: Guidance satisfaction*

1. What do you think of the guidance at work by your supervisor?
   1. Why are you satisfied or not satisfied?
2. What do you like/ not like about the supervision?
3. Why is your supervisor a good or not a good supervisor?
   1. What is your supervisor doing right/wrong?
4. What kind of qualities/skills does a perfect supervisor have?
   1. Which qualities should a supervisor have to guide you at the workplace?
   2. Which qualities should a supervisor not have to guide you at the workplace?

*Topic 3: Change in guidance after the ‘Mentorwijs’ training*

1. Has the guidance changed/improved in recent months?
2. What kind of improvements/changes did you notice?

*Topic 4 & 5: Fit between knowledge and skills and the demands of the job and confidence performance of the job*

1. What do you think about your work tasks?
   1. Do you know how to perform your work tasks?
   2. Do you feel confident that you can perform your work tasks in the right way?
   3. Can you perform your work tasks independently?
2. Does your supervisor help you with performing your work tasks?
   1. How does your supervisor help you?
   2. Made your supervisor adjustments at work so that you perform you work tasks?
3. If you are unable to complete a work task, what do you do?
   1. Do you ask your supervisor for help? Are you able to ask for help?
   2. Would you like extra help from your supervisor? And what kind of help?
4. Do you ever have problems at work?
   1. What do you do in case there are problems? How do you solve these problems?
   2. Can you/do you go to your supervisor?
   3. Does your supervisor help you with solving problems? And in which way?
   4. Does your supervisor notice if there are problems?
5. Do you have the opportunity to learn new things and/or to grow at work?
   1. Are your work tasks easy? Or too difficult?
   2. Are your work tasks complicated? Or too monotonous?
   3. Do you have the ability to perform other work tasks?

*Topic 6: Position in the company*

1. Do you feel that you are taken seriously at work?
   1. Do you feel that you are appreciated/accepted at work?
2. Do you feel that you are equal to your colleagues?
   1. Are you the only employee in the company with work disability?
   2. Do you feel you are treated differently than your colleagues at work?
   3. Do you feel that your colleagues or your supervisor listens to you?
