# Supplementary material for: “I noticed that when I have a good supervisor, it can make a Lot of difference.” A Qualitative Study on Guidance of Employees with a Work Disability to Improve Sustainable Employability
Source: J Occup Rehabil. 2022 Sep 6;33(1):201–12. doi: 10.1007/s10926-022-10063-6 (PMC10025227; doi:10.1007/s10926-022-10063-6)
Supplement: Supplementary file 2 — Supplementary Material 2 [file 10926_2022_10063_MOESM2_ESM.docx]

**Codebook**

| **Theme** | **Sub-code** | **Codes** |
| --- | --- | --- |
| Work tasks and conditions can facilitate or hinder sustainable employability | Facilitators of work(tasks) and working conditions for sustainable employability | Fun at work |
|  |  | Work is routine/easy |
|  |  | Enjoyable atmosphere |
|  |  | To be able to perform work tasks independently |
|  |  | No workload at work |
|  |  | A lot of freedom at work |
|  |  | Employees want to work for a long period of time |
|  |  | A lot of variation in work tasks |
|  |  | Work tasks are easy |
|  |  | A lot of structure |
|  |  | Adjustments made for employee |
|  |  | Perform work tasks step-by-step |
|  | Barriers of work(tasks) and working conditions for sustainable employability | Work sometimes energy consuming |
|  |  | Work tasks sometimes boring |
|  |  | Work is monotonous |
|  |  | A lot of work hours |
|  |  | Hard working |
|  |  | Difficult working conditions |
|  |  | Cleaning up mess of others |
|  |  | Difficult working conditions |
|  |  | Not have proper work clothes |
|  |  | Work tasks with long concentration difficult |
|  | Needs for work(tasks) and working conditions | Employee wants to feel useful at work |
|  |  | Employee wants more structure at work |
|  |  | Employee needs more variation |
|  |  | Employee does not want repetitive work tasks |
|  |  | Employee wants more responsibility |
|  |  | Employee wants work with societal relevance |
| Relationships among employees and with supervisors can affect sustainable employability | Positive relationship with colleagues | Good collaboration |
|  |  | Get on well together |
|  |  | Equality at the workplace |
|  |  | No hierarchy |
|  |  | Colleagues with a lot of experience |
|  |  | Never conflicts |
|  |  | Colleagues want to help each other |
|  | Positive relationship with supervisor | A lot of understanding from supervisor/organization |
|  |  | Solving conflicts through talking |
|  |  | Conflicts quickly resolved |
|  |  | After training more collaboration and more considerate of each other |
|  | Negative relationship with colleagues | Sometimes no equality |
|  |  | Arguing or irritations with each other |
|  |  | Gossip among each other |
|  |  | Not getting along with each other |
|  |  | Conflicts occur |
|  | Negative relationship with supervisor | Hates supervisor |
|  |  | Supervisor must not treat employee like a child |
|  |  | Conflicts with supervisor |
| A desire for new opportunities and challenges | Desires for new opportunities and challenges | Employees wants challenging work |
|  |  | Employee wants to be able to grow |
|  |  | Employee wants to learn new work tasks |
|  | Opportunities to learn and to get new challenges | Work is educational |
|  |  | Work is challenging |
|  |  | Opportunities to learn new work tasks |
|  |  | Making mistakes is allowed |
|  |  | There are growth opportunities |
|  |  | Learning step-by-step |
|  |  | Employee do not often get chances to grow/learn |
| A need for supervisor skills to facilitate sustainable employability | Communication; positive points | Pleasant way of communicating |
|  |  | Clear explanation of supervisor |
|  |  | After training better communication |
|  | Communication; negative points | Not talking about employees, but with employees |
|  |  | Communicate more clearly |
|  |  | At the beginning more explanation about work tasks |
|  |  | Need clear communication about work task |
|  |  | No communication between supervisors |
|  |  | Communication contradictory |
|  |  | Not a pleasant way of communicating |
|  |  | Need of a chat now and then |
|  |  | Little contact with supervisor |
|  |  | Promises not kept |
|  |  | Do not receive compliments |
|  | Attitude; positive points | Supervisor is friendly |
|  |  | Supervisor is reliable |
|  |  | Employee is taken seriously |
|  |  | Supervisor considerate employees |
|  |  | Important that a supervisor is patient |
|  |  | After training supervisor became more relaxed |
|  |  | After training supervisor kept closer eye on employee and more collaboration |
|  | Attitude; negative points | Employees are not taken seriously |
|  |  | Supervisor need to be more considerate with employees |
|  |  | Employees expect more trust from supervisor |
|  |  | Supervisor is negative/not fun |
|  |  | Supervisor is inpatient |
|  | Listening; positive points | Supervisors listen well |
|  | Listening; negative points | Supervisor does not listen well |
|  |  | As employee very little to say |
|  |  | Criticism is cut off |
|  |  | Supervisor must listen more to opinion of employees |
|  |  | Supervisors must be available to listen |
|  | Dealing with problems; positive points | With a problem to the supervisor |
|  |  | Problem picked up by supervisor |
|  |  | Supervisor available to talk about problems |
|  |  | Supervisor must be willing to help employee |
|  | Dealing with problems; negative points | Problems not addressed by supervisor |
|  | Availability of help; positive points | Help always available |
|  |  | Asking questions always available |
|  |  | Supervisor notices when help is needed |
|  | Availability of help; negative points | Due to pressure at work not possible or waiting to ask questions |
|  |  | Supervisor lack of time |
|  |  | Sometimes in need of more help |
|  |  | On own initiative asking for help |
|  |  | Supervisor should be more available |
|  |  | Supervisor must be available to ask questions |
|  | Appreciation; positive points | Appreciation for work |
|  |  | Receiving compliments for work |
|  |  | Supervisor provides (positive) feedback |
|  |  | More motivation through receiving compliments |
|  |  | After training more compliments |
|  | Appreciation; negative points | A lack of appreciation |
|  |  | Need more appreciation |
|  |  | Need more compliments |
|  | Mentorwijs | Did not notice any change after the training in guidance of supervisors |
|  |  | Remained satisfied about supervision after training |
|  |  | Training was informative for supervisor |
|  |  | Noticed a difference after the training |
